# Surgical treatment of a penoscrotal massive localized lymphedema: Case report

**DOI:** 10.1016/j.ijscr.2019.05.022

**Published:** 2019-05-14

**Authors:** Rodolfo Costa Lobato, Rafael Ferreira Zatz, Wilson Cintra Junior, Miguel Luiz Antonio Modolin, Alex Chi, Yanessa Katiana Van Dunem Filipe de Almeida, Rolf Gemperli

**Affiliations:** Plastic Surgery Department, Hospital das Clinicas, University of São Paulo, Brazil

**Keywords:** Urology, Urological surgical procedures, Lymphedema, Penis

## Abstract

**Introduction:**

Massive localized lymphedema is an aggressive type of lymphedema that causes great functional impairment for the patient, depriving from one’s basic life activities. The treatment of this type of lesion is eminently surgical, requiring ablative surgery (complete surgical resection of the lesion), but the possible techniques not always provide a good functional result.

**Presentation of case:**

We reported a case of a penoscrotal massive lymphedema treated by our Body Contour Group/Plastic surgery department of our institute. We performed the resection of the giant penoscrotal lesion, used a posterior scrotal flap for defect’s reconstruction and a split-thickness skin graft for penis’ body reconstruction, closed with Z-plasty.

**Discussion:**

Contrary to what the literature says, we prefer to use the split-thickness skin graft to reconstruct the penis’ body in these cases, against local flaps. According to our experience with some similar cases, this technique provides a better functional result once it allows the penis to a better expansion during erection. The key maneuver to avoid contracture of the graft and retraction of the penis is to perform a broken line suture (Z-plasty) in the topography of the median raphe.

**Conclusion:**

In cases of penoscrotal massive lymphedema, the treatment’s option with better results is the surgical one. The use of a scrotal flap associated with split-thickness skin graft for penis provides good aesthetic and functional outcomes.

## Highlights

• Massive penoscrotal lymphedema is an aggressive type of lymphedema with great functional impairment for the patient.
• The treatment of this type of lesion is eminently surgical, requiring ablative surgery.
• Our technique consists of a posterior scrotal flap and a split-thickness skin graft for penis’ body reconstruction.
• The key maneuver to avoid retraction of the penis is Z-plasty in the topography of the median raphe.

## 1 Introduction

Lymphedema is the lymph’s accumulation in the interstitial space due to an obstruction or rupture of the lymphatic ducts. Primary lymphedema refers to agenesis or congenital malformation of the lymphatic system, while the secondary cases may have several etiologies, like trauma, local radiation, lymph node dissection, among others [[1], [2], [3]].

The clinical presentation commonly is mild, but yet with significant interference in the quality of life. In contrast, a severe presentation called massive localized lymphedema (MLL) causes great functional impairment for the patient, depriving from one’s basic life activities [1,2]. It’s an acquired chronic condition of idiopathic etiology and with great association with morbid obesity, occurring mainly on abdominal wall and thighs, being less frequent in the scrotal region [3].

Scrotal massive lymphedema, also known as scrotal elephantiasis, is a rare event. The main etiology is the occurrence of venereal lymphogranuloma or penoscrotal filariasis [3].

Mostly, clinical measures are not able to solve the problem and functional surgeries are not indicated to these cases, once the skin and subcutaneous are at an advanced degree of involvement. The treatment of this type of lesion is eminently surgical, requiring ablative surgery (complete surgical resection of the lesion) but many of these cases have bad outcomes, without good functional results [[4], [5], [6], [7]].

The objective of this study was to report the surgical strategies to treat a localized massive lymphedema of the penoscrotal region and show de long-term follow up, with good outcomes. This work has been reported in line with the SCARE criteria [8].

## 2 CASE PRESENTATION

A 60-year-old man, with no comorbidities and living in a rural area free of Filariasis, begun a follow-up with the Body Contour Group (Plastic Surgery Department) of our institute in 2016. He reported that the scrotum began to swell in 2009, slowly and progressively, to a massive injury, depriving him of physical and sexual activities. Although he denied urogenital infections, he reported that he had recurrent episodes of scrotal erysipelas.

**Fig. 1:**
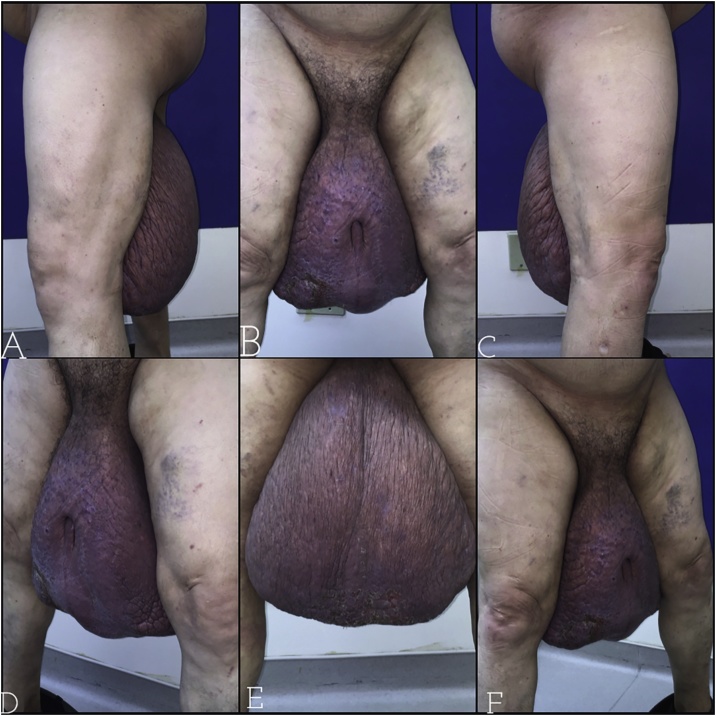
Preoperative - A/C: Side views; B: Frontal view; D/F: 45° views; E: Back view.

On physical examination, it was evident a massive lymphedema of the scrotum, approximately 20 cm x 30 cm x 40 cm, with a buried penis in the mass and non-palpable testicles. The skin of the scrotum presented with increased thickness and areas of peeling, hardened when palpated. A BMI of 27 was calculated. (Fig. 1).

### 2.1 Preoperative

**Fig. 2:**
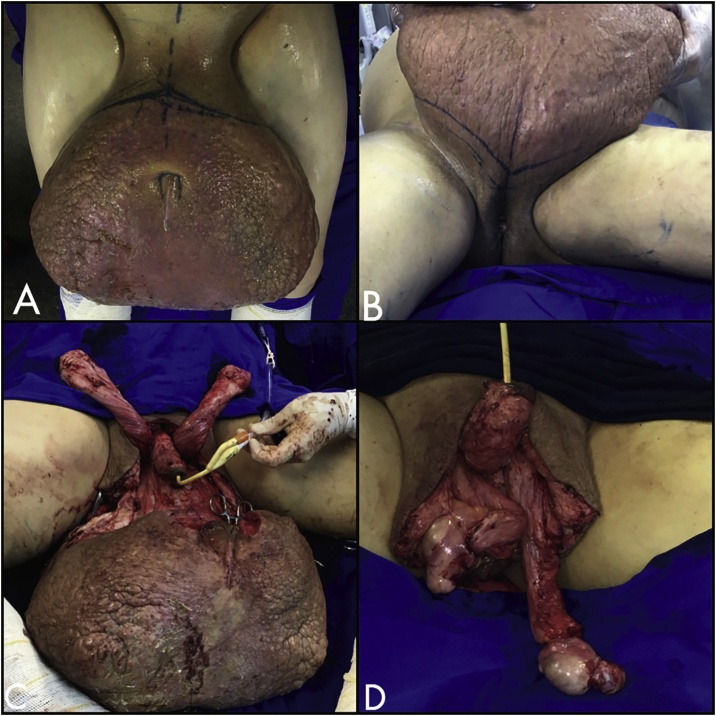
A-B: Preoperative surgical demarcation. C: Dissection of the penis and spermatic cord; D: Orchidopexy of the right testicle.

The patient’s preoperative prepare was done with a cleaning of the inguinoscrotal region for three consecutive days with chlorhexidine, 3 times a day. The patient, when in decubitus, was maintained with continuous elevation of the lower limbs. Ciprofloxacin was used as prophylactic antibiotic. The preoperative surgical demarcation consisted of marking the midline and the transition between healthy skin and diseased skin, circumferentially, aiming to involve the entire areas with lymphedema. (Fig. 2)

In addition, an anesthetist and a cardiologist evaluated the patient in preoperative and considerated his surgical risk as moderate, indicating general anesthesia, thrombosis prophylaxis in postoperative and measurement of myocardial necrosis markers, as recommended in Novo et al. [9].

### 2.2 Surgical technique

An incision was made in the marked area, beginning with the supra-pubic region; a careful dissection was carried out within the infiltrated and hardened tissue that occupies the entire mass; as the dissection progressed, the skin incision is continued throughout the circumference of the mass. Meticulous dissection allows preservation of the penile body and the elements of the spermatic cord, resecting the skin of the penis superficial to the Buck's fascia.

**Fig. 3:**
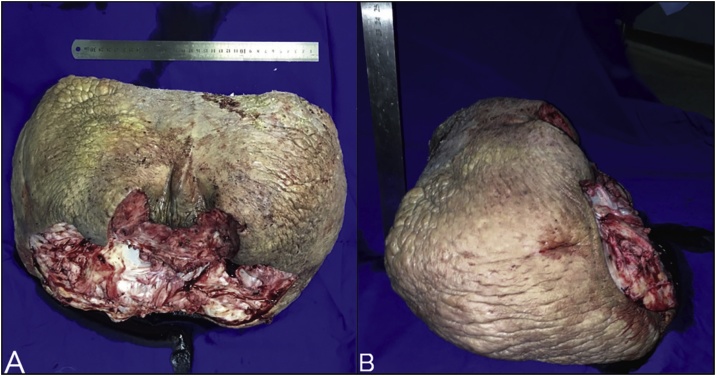
A/B: The resected lesion, frontal and side view.

**Fig. 4:**
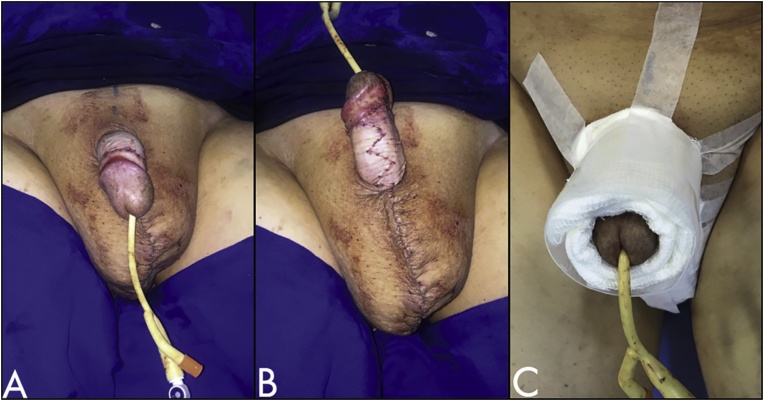
Postoperative – A/B: Immediate postoperative – partial skin graft of the penile body with posterior Z-plasty to avoid scar contracture and reconstruction of the scrotum with the posterolateral skin flaps. C: The closure done to assure good integration of the skin graft.

During the resection of the lesion (Fig. 3) (sent to anatomopathological study), we preserved two posterolateral healthy skin flaps in the perineal region, which were used for reconstruction of the scrotum. The tunica albuginea was opened, to avoid hydrocele, and bilateral orchidopexy was made to avoid testicular torsion. (Fig. 2). Subsequent coverage with the aforementioned flaps was then performed (Fig. 4).

For penile coverage, split-thickness skin graft (removed from the left thigh) was fixed between the glans and the base of the penis. To avoid contracture of the graft and retraction of the penis, a broken line suture (Z-plasty) was used in the topography of the median raphe.

### 2.3 Postoperative

The skin graft was kept occluded with a dressing adapted for medium compression and held for 5 days to avoid local traumas and consequent loss of it. The bladder catheter was maintained for the same period and the hygiene of the genital area was performed daily by the nursing team, avoiding manipulation by the patient. Graft opening was performed on the 5th postoperative day, with total graft integration. The patient was discharged on the 7th postoperative day, with local care guidelines. Anatomopathological exam confirmed chronic lymphedema. The mass weighted 9,9 kg.

**Fig. 5:**
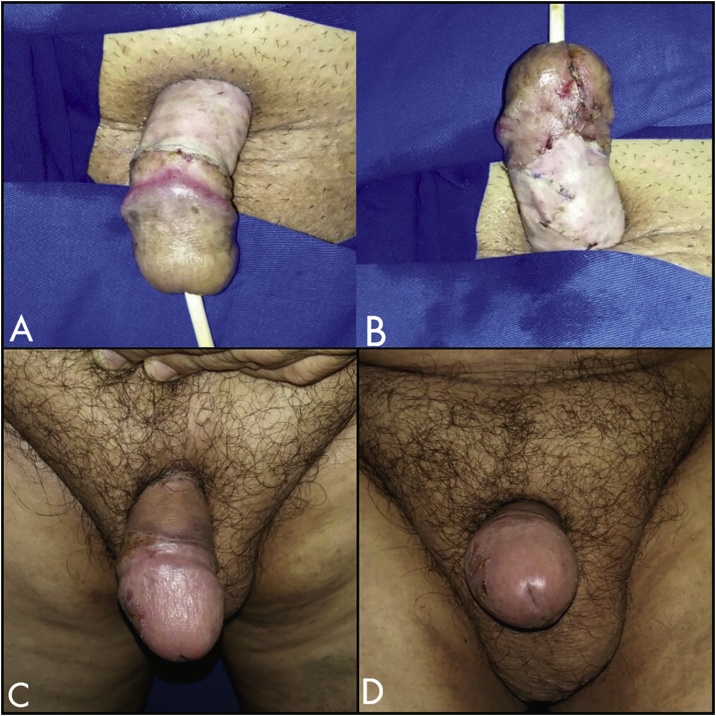
A: 7-days postoperative; B: 20-month follow-up.

The follow-up was kept for 20 months (Fig. 5). The patient regained sexual and physical activities and he had not had new episodes of erysipelas.

## 3 Discussion

It is presented a case of idiopathic massive localized penoscrotal lymphedema. In the literature, MLL cases are most related to obese patients and usually are seen at the abdomen or lower limbs [3,10]. This case contrast with that evidence, since it involved the penoscrotal region and the patient never had a BMI higher than 27.

Clinical measures were the main treatment over 6 years, without improvement in the quality of life of this patient. The poor response of the clinical treatment was the main motivation for the surgical treatment. Functional operations (lymph node transplantation and lymphaticovenous anastomosis) were not indicated since it was an advanced case, with a thick and fibrotic skin and subcutaneous.

Hence, the radical and gentle resection was the chosen procedure. Due to a lack of cleavage plane during the resection, it was difficult to preserve the genital structures within the mass [[11], [12], [13]]. Fortunately, iatrogenic damage was avoided, and the outcome was satisfying. Sexual and physical rehabilitation was achieved. At the last follow-up, the patient did not relate any new episodes of erysipelas.

Currently, with the upcoming of the physiological techniques, the surgical treatment of the lymphedema has been more discussed. However, few are the articles approaching the penoscrotal lymphedema treatment, with low level of evidence. There`s a belief of benefit from lymphatic anastomosis and lymph node transplantation on mild to intermediate cases [11,13,14]. Conversely, most of publications relate the treatment of advanced cases, when the radical resection is the only and gold standard procedure done [4,[11], [12], [13], [14], [15], [16], [17]].

Rewarding results of the radical resection were published, even with different reconstruction options. The scrotal coverage is similar in the most publications, as well as the presented case: use of postero-lateral healthy skin flaps. Whereas, the penile body skin reconstruction differs according to the author, despite the common successful result in long term follow-up, with markedly increase on patient`s quality of life. Irdam et al. and Torio-Padron et al. exposed the use of adjacent healthy skin to penile coverage [15,17,18]. Local pediculated flaps are other satisfying reconstructive options [12,13,16,19].

## 4 Conclusion

Our Body Contour Group believe, that for penis’ body reconstruction, split-thickness skin graft, as seen in Modolin et al. 2006, is a good option, as presented in this case [4]. Larger series expose the same encouraging results of radical resection and reconstruction with split-thickness skin grafts (Modified Charles Procedure) [4].

## Conflicts of interest

All the authors state they have nothing to disclose/No conflict of interest.

## Funding sources

The research did not receive any specific funding from funding agencies in the public, commercial, or not-for-profit sectors.

## Ethical approval

As a Case Report, this study was exempted of ethical approval by the “**Comissão de Ética para Análise de Projetos de Pesquisa – CAPPesq**” (**Ethics committee to research projects analysis**).

In our institution, ethnical approval is not necessary to do this study.

## Consent

Written informed consent was obtained from the patient for publication of this case report and accompanying images. A copy of the written consent is available for review by the Editor-in-Chief of this journal on request.

## Author contribution

**Rodolfo Costa Lobato**: Conceptualization; Data curation; Formal analysis; Roles/Writing - original draft.

**Rafael Ferreira Zatz**: Conceptualization; Data curation; Formal analysis; Roles/Writing - original draft.

**Wilson Cintra Junior**: Conceptualization; Methodology; Project administration; Supervision; Validation; Writing - review & editing.

**Miguel Luiz Antonio Modolin**: Conceptualization; Methodology; Project administration; Supervision; Validation; Writing - review & editing.

**Alex Chi**: Conceptualization; Data curation; Formal analysis; Roles/Writing - original draft.

**Yanessa Katiana Van Dunem Filipe de Almeida**: Conceptualization; Data curation; Formal analysis; Roles/Writing - original draft.

**Rolf Gemperli**: Conceptualization; Methodology; Project administration; Supervision; Validation; Writing - review & editing.

## Registration of research studies

As a Case Report, not registered.

## Guarantor

Wilson Cintra Junior; Rolf Gemperli.

## Provenance and peer review

Not commissioned, externally peer-reviewed.
